# Exploring Nonlinear Diffusion Equations for Modelling Dye-Sensitized Solar Cells

**DOI:** 10.3390/e22020248

**Published:** 2020-02-21

**Authors:** Benjamin Maldon, Ngamta Thamwattana, Maureen Edwards

**Affiliations:** 1 School of Mathematical and Physical Sciences, University of Newcastle, Callaghan NSW 2308, Australia; natalie.thamwattana@newcastle.edu.au; 2 School of Mathematics and Applied Statistics, University of Wollongong, Wollongong NSW 2522, Australia; maureen@uow.edu.au

**Keywords:** dye-sensitized solar cells, electron density, efficiency, nonlinear diffusion, Lie symmetry

## Abstract

Dye-sensitized solar cells offer an alternative source for renewable energy by means of converting sunlight into electricity. While there are many studies concerning the development of DSSCs, comprehensive mathematical modelling of the devices is still lacking. Recent mathematical models are based on diffusion equations of electron density in the conduction band of the nano-porous semiconductor in dye-sensitized solar cells. Under linear diffusion and recombination, this paper provides analytical solutions to the diffusion equation. Further, Lie symmetry analysis is adopted in order to explore analytical solutions to physically relevant special cases of the nonlinear diffusion equations. While analytical solutions may not be possible, we provide numerical solutions, which are in good agreement with the results given in the literature.

## 1. Introduction

Given the increasing demand for affordable renewable energy, solar cell research seeks ways to alleviate the relatively high cost of materials while maintaining high efficiency. In 1991, O‘Regan and Grätzel [1] introduced dye-sensitized solar cells (DSSCs) and demonstrated their potential to provide an alternative solution for solar cell technology. In particular, expansive research areas on determining new low cost materials, optimal structures, and configurations of the device have emerged to maximise the performance and efficiency of DSSCs.

**Figure 1:**
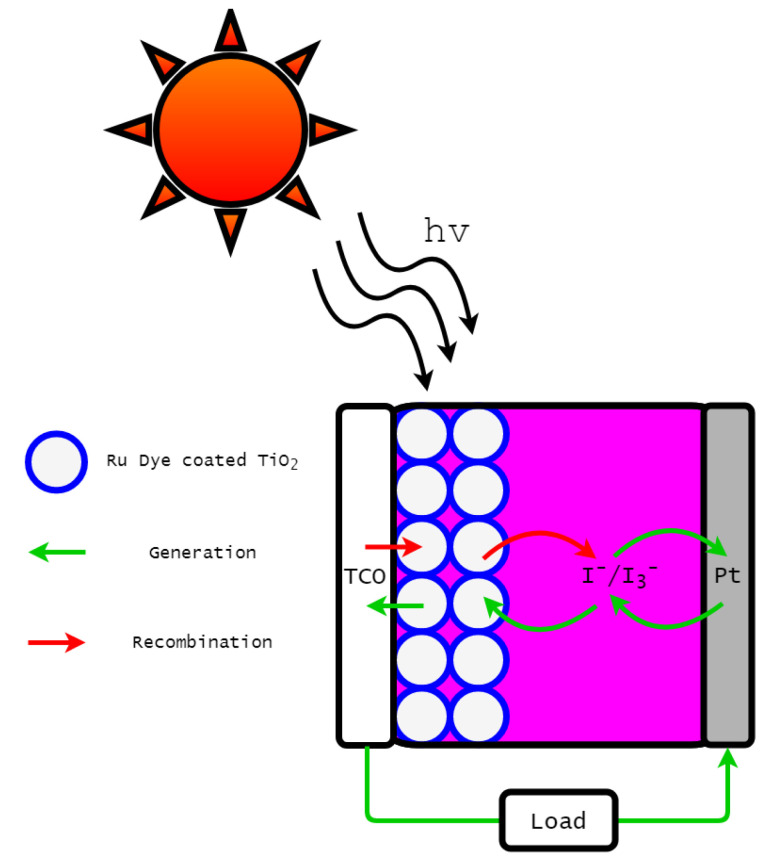
Diagram of a functioning DSSC.

A DSSC is composed of four primary components; a photosensitive dye, a nano-porous semiconductor, an electrolyte couple, and a counter electrode. Traditionally, the Ruthenium (II) photosensitive dye, the nano-porous semiconductor ${\mathrm{TiO}}_{2}$, the iodide-triiodide electrolyte couple $I^{-}$-$I_{3}^{-}$, and a platinised counter electrode are typically used in DSSCs [1,2]. As depicted in Figure 1, sunlight excites dye molecules to a high energy state. This provides energy for the dye to donate an electron to the nano-porous semiconductor (a process known as electron injection), which leaves the DSSC to power the load. Electrons are reintroduced through the counter electrode, which returns electrons back to the photosensitive dye through the redox electrolyte couple.

To determine the key performance parameters of a DSSC (including its efficiency), it is necessary to derive the current-voltage relationship. To obtain this, one modelling approach is to use a drift-diffusion model based on studies of junction solar cells [3,4]. However, the electric field that is usually present in typical junction solar cells is considered to have an insignificant influence on electron transport in DSSCs. As such, it can be argued that drift-diffusion models may not be appropriate for modelling DSSCs [5]. Alternatively, Södergren et al. proposed a diffusion equation for the electron density in the nano-porous semiconductor of DSSCs [2]. Since this model is based on a linear, second order ordinary differential equation, the model admits analytical solutions for the electron density. Expressions for the current-voltage relationship for DSSCs can therefore be derived analytically [2]. Nevertheless, several terms in the diffusion model are inherited from the drift-diffusion model of junction solar cells (such as the Beer–Lambert law for electron generation).

Since the study of Södergren et al. in 1994 [2], the diffusion model for DSSCs has undergone significant development. In particular, in 1996, Cao et al. added time-dependence to the diffusion equation and introduced a nonlinear electron diffusion term into their partial differential equation (PDE) [6]. While the model proposed by Cao et al. [6] was only concerned with the electron density in the nano-porous semiconductor, Papageorgiou et al. [7] considered the effect of electrolyte concentrations, to allow understanding of the distribution of electron density in DSSCs beyond the nano-porous semiconductor.

In 2006, Anta et al. [8] revisited the electron diffusion equation given in [6] and introduced a nonlinear term for loss of electrons at material interfaces, known as electron recombination. Later, Barnes and O’Regan [9] introduced modified recombination kinetics for the linear diffusion equation. In 2010, Bisquert and Mora-Seró studied [10] the electron diffusion equation with a constant diffusion coefficient and a modified recombination term to gain insight into the electron diffusion length. In 2011, Andrade et al. [11] combined the linear PDE for the electron density in the nano-porous semiconductor given by Cao et al. [6] and the electron density model in the electrolyte solution given by Papageorgiou et al. [7] to produce a new system of linear PDEs. These equations were also numerically investigated by Gacemi et al. [12].

In the following subsection, we briefly state the nonlinear PDE used to model the diffusion of electrons in the nano-porous semiconductor of DSSCs. Furthermore, the relationships between the electron density, the current-voltage profile, and the efficiency are given in Section 1.2.

### 1.1. Diffusion Equation

Given a DSSC of thickness *d*, let n(x,t) be the electron density at depth x∈[0,d] and time t≥0. Here, x=0 denotes the location of the transparent conductive oxide (TCO), and x=d is the location of the counter electrode of the device. From [8], the electron density satisfies:

(1) $$\frac{\partial n}{\partial t}=D_{0}\frac{\partial }{\partial x}\left[{\left(\frac{n}{n_{eq}}\right)}^{m}\frac{\partial n}{\partial x}\right]+\varphi \alpha_{ab}e^{-\alpha_{ab}x}-k_{R}{\left(\frac{n}{n_{eq}}\right)}^{m}\left(n\left(x,t\right)-n_{eq}\right),$$

where $D_{0}$ is the diffusion coefficient, $n_{eq}$ is the dark equilibrium electron density, φ is the incident photon flux, $\alpha_{ab}$ is the absorption coefficient of the Ruthenium (II) dye, $k_{R}$ is the recombination coefficient, and *m* is the diffusion order. In this paper, we refer to Equation (1) as the electron diffusion equation.

To prescribe boundary conditions to Equation (1), we assume a Boltzmann approximation for the density at the TCO substrate (which is valid for nanoporous semiconductors with fast electron transfer rates [8,10,13]) and that the current density vanishes at the counter electrode [14,15]. This yields the boundary conditions:

(2) $$n\left(x,0\right)=n_{eq}e^{\frac{qV}{m_{I}k_{B}T}},\;n\left(0,t\right)=n_{eq}e^{\frac{qV}{m_{I}k_{B}T}},\;\frac{\partial n}{\partial x}{|}_{x=d}=0,$$

where *q* is the standard electron charge, *V* is the applied bias voltage of the DSSC, $m_{I}$ is the diode ideality factor, $k_{B}$ is Boltzmann’s constant, and *T* is the temperature of the DSSC. Short-circuit conditions may therefore be found under the special case V=0. For open-circuit conditions, we replace the Dirichlet boundary condition at x=0 with the Neumann boundary condition:

$$\frac{\partial n}{\partial x}{|}_{x=0}=0.$$

To nondimensionalise Equation (1), we use the same scaling parameters as of Cao et al. [6], namely:

(3) $$\bar{n}=\frac{n}{n_{eq}},\;\bar{x}=\frac{x}{d},\;\bar{t}=\frac{D_{0}t}{d^{2}},$$

with the additional nondimensional parameter $\omega =\frac{qV}{m_{I}k_{B}T}$, which depends on the value of applied voltage *V*. The nondimensional equation is therefore given by:

(4) $$\frac{\partial \bar{n}}{\partial \bar{t}}=\frac{\partial }{\partial \bar{x}}\left({\bar{n}}^{m}\frac{\partial \bar{n}}{\partial \bar{x}}\right)+\mu e^{-\nu \bar{x}}-\xi {\bar{n}}^{m}\left(\bar{n}-1\right),$$

where our nondimensional parameters are $\mu =\frac{d^{2}\varphi \alpha_{ab}}{D_{0}n_{eq}}$, $\nu =\alpha_{ab}d$ and $\xi =\frac{k_{R}d^{2}}{D_{0}}$. Dropping the bar notation, the boundary and initial conditions for $V\neq V_{oc}$ become:

$$n\left(x,0\right)=e^{\omega },\;n\left(0,t\right)=e^{\omega },\;\frac{\partial n}{\partial x}{|}_{x=1}=0,$$

and the conditions for open-circuit are given by:

$$n\left(x,0\right)=e^{\omega_{oc}},\;\frac{\partial n}{\partial x}{|}_{x=0}=0,\;\frac{\partial n}{\partial x}{|}_{x=1}=0,$$

where $\omega_{oc}=\frac{qV_{oc}}{m_{I}k_{B}T}$. In Table 1, the values of physical parameters of DSSCs found in Anta et al. [8] and Gacemi et al. [12] are presented. These values are used to compute our nondimensional parameters μ, ν, and ξ, the values of which are also given in Table 1. We comment that this paper only considers m≥0. For the special case of m=0, Equation (4) reduces to a linear PDE for which analytical solutions can be found, as shown in Section 2. Further, Anta et al. [8] studied the special cases of m=1,2,3, and Cao et al. [6] considered the case of m=1.

### 1.2. Current-Voltage Characteristics

Here, we give the relationship between the electron density, the current, and the voltage and the formula for calculating the efficiency of DSSCs.

First, the current density *J* as a function of the electron density *n* is given by:

(5) $$J=\frac{qD_{0}n_{eq}}{d}\frac{\partial n}{\partial x}{|}_{x=0}.$$

Similarly, the short-circuit current density $J_{sc}$ is given by:

$$J_{sc}=\frac{qD_{0}n_{eq}}{d}\frac{\partial n_{sc}}{\partial x}{|}_{x=0},$$

where $n_{sc}$ is the electron density under short-circuit conditions (V=0). Given the short-circuit current density $J_{sc}$, we apply the diode equation for the voltage-dependent current density J(V) [2]:

(6) $$J\left(V\right)=J_{sc}-J_{0}\left(e^{\frac{qV}{m_{I}k_{B}T}}-1\right),$$

where $J_{0}$ is the dark saturation current density [2], given by:

$$J_{0}=\frac{qD_{0}n_{eq}}{d}\tanh\left(\frac{d}{\sqrt{\frac{D_{0}}{k_{R}}}}\right).$$

By definition of the open-circuit voltage $V_{oc}$, we have $J(V_{oc})=0$. Therefore, the open-circuit voltage $V_{oc}$ is obtained from [18]:

(7) $$V_{oc}=\frac{m_{I}k_{B}T}{q}\ln\left(\frac{J_{sc}}{J_{0}}+1\right).$$

### 1.3. Efficiency

To calculate efficiency, we first compute the power output of the DSSC by the standard formula P=JV. Writing J=J(V), maximising *P* is traditionally done over *V* to obtain the maximum power point $P_{max}=J\left(V_{max}\right)V_{max}$. The efficiency of the DSSC, η, is then calculated from:

(8) $$\eta =\frac{P_{max}}{P_{i}},$$

where $P_{i}$ is the total available power from sunlight, a value typically taken as 1000 $Wm^{-2}$ [9,16,17].

In the following section, we consider a special case of Equation (4) where we assume m=0. This assumption leads to a linear diffusion equation for which exact analytical solutions are obtained as shown in Section 2.1 and Section 2.2 for bias voltage and open-circuit models, respectively. In Section 3, we investigate the case of m≥0 and use Lie symmetry techniques to explore the possibility of finding new analytical solutions to the nonlinear diffusion equations. Finally, Section 4 presents numerical results of the nonlinear model together with some discussion of the results and potential research directions in this area.

## 2. Analytical Solution for the Linear Electron Diffusion Equation

Assuming m=0, Equation (4) reduces to a linear PDE of the form:

(9) $$\frac{\partial n}{\partial t}=\frac{\partial^{2}n}{\partial x^{2}}+\mu e^{-\nu x}-\xi \left(n(x,t)-1\right).$$

This special case readily admits an analytical solution, using a standard technique as shown in [19]. Since Equation (9) is linear, the solution can be expressed as:

n(x,t)=u(x,t)+f(x),

where *f* is a particular solution to:

$$\frac{d^{2}f}{dx^{2}}+\mu e^{-\nu x}-\xi \left(f\left(x\right)-1\right)=0.$$

Using Equation (9), we find that u(x,t) must satisfy:

$$\frac{\partial u}{\partial t}=\frac{\partial^{2}u}{\partial x^{2}}-\xi u\left(x,t\right).$$

The boundary conditions applied for *f* are imposed to allow separation of variables for finding *u*, according to whether the model is under open-circuit conditions (resulting in two Neumann boundary conditions) or a given voltage bias *V* (resulting in a Dirichlet boundary condition and a Neumann boundary condition).

### 2.1. Short-Circuit Conditions

Under the standard bias voltage conditions (for which $V\neq V_{oc}$), we solve Equation (9) under the conditions:

$$n\left(x,0\right)=e^{\omega },\;n\left(0,t\right)=e^{\omega },\;\frac{\partial n}{\partial x}{|}_{x=1}=0.$$

Here, we find the particular solution $f_{v}$ to be given by:

(10) $$f_{v}\left(x\right)=1+Ae^{\sqrt{\xi }x}+Be^{-\sqrt{\xi }x}-\frac{\mu }{\nu^{2}-\xi }e^{-\nu x},$$

where the constants of integration *A* and *B* are given by:

$$\begin{array}{ccc} A & = & -\frac{\mu \nu e^{\sqrt{\xi }-\nu }+\xi^{\frac{3}{2}}\left(e^{\omega }-1\right)-\sqrt{\xi }\left(e^{\omega }\nu^{2}-\nu^{2}+\mu \right)}{\sqrt{\xi }\left(\nu^{2}-\xi \right)\left(e^{2\sqrt{\xi }}+1\right)},\\ B & = & \frac{e^{\sqrt{\xi }-\nu }\left[e^{\sqrt{\xi }+\nu +\omega }\left(\sqrt{\xi }\nu^{2}-\xi^{\frac{3}{2}}\right)+\mu \nu +e^{\sqrt{\xi }+\nu }\left(\xi^{\frac{3}{2}}+\sqrt{\xi }\left(\mu -\nu^{2}\right)\right)\right]}{\sqrt{\xi }\left(\nu^{2}-\xi \right)\left(e^{2\sqrt{\xi }}+1\right)}. \end{array}$$

Finally, using a separation of variables approach, *u* is given by:

$$u\left(x,t\right)=\sum_{k=0}^{\infty }C_{k}\sin\left(\frac{(2k+1)\pi }{2}x\right)e^{-\left[{\left(\frac{(2k+1)\pi }{2}\right)}^{2}+\xi \right]t},$$

where the constants $C_{k}$ are given by:

$$C_{k}=2\int_{0}^{1}\sin\left(\frac{(2k+1)\pi }{2}x\right)\left[e^{\omega }-f_{v}\left(x\right)\right]dx.$$

In summary, the analytical solution for Equation (9) with a bias voltage $V\neq V_{oc}$ is given by:

(11) $$n\left(x,t\right)=1+Ae^{\sqrt{\xi }x}+Be^{-\sqrt{\xi }x}-\frac{\mu }{\nu^{2}-\xi }e^{-\nu x}+\sum_{k=0}^{\infty }C_{k}\sin\left(\frac{(2k+1)\pi }{2}x\right)e^{-\left[{\left(\frac{(2k+1)\pi }{2}\right)}^{2}+\xi \right]t}.$$

**Figure 2:**
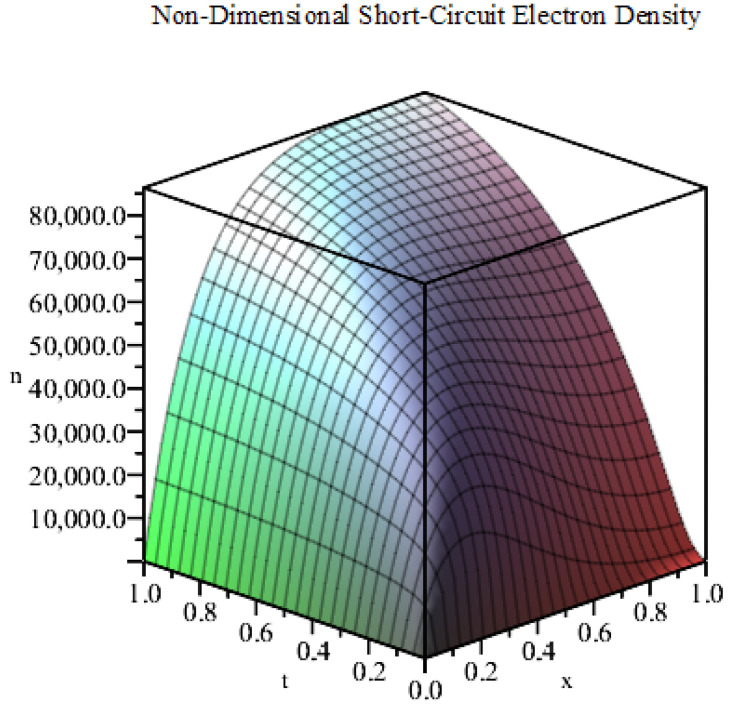
Plot of *n* against *x* and *t* (m=0, short-circuit conditions).

In Figure 2, we plot the exact solution (11) under short-circuit conditions (by setting ω=0) using the data given in Table 1 for the first 100 terms of the infinite series in MAPLE. From the figure, we can see that the electron density rises quickly from its dark equilibrium due to the influence of the exponential source term of electron generation. The electron density continues to increase until it reaches the steady-state, as expected for a linear diffusion problem.

### 2.2. Open-Circuit Model

For the open-circuit model, we solve Equation (9) under the following conditions:

$$n\left(x,0\right)=e^{\omega_{oc}},\;\frac{\partial n}{\partial x}{|}_{x=0}=0,\;\frac{\partial n}{\partial x}{|}_{x=1}=0.$$

Using the same approach as before, we find the particular solution $f_{oc}$ for open-circuit conditions as:

(12) $$f_{oc}\left(x\right)=1+A_{oc}e^{\sqrt{\xi }x}+B_{oc}e^{-\sqrt{\xi }x}-\frac{\mu }{\nu^{2}-\xi }e^{-\nu x},$$

where the constants of integration are given by:

$$A_{oc}=-\frac{\mu \nu (e^{\sqrt{\xi }-\nu }-1)}{\sqrt{\xi }\left(\nu^{2}-\xi \right)\left(e^{2\sqrt{\xi }}-1\right)},\;B_{oc}=\frac{\mu \nu e^{\sqrt{\xi }}\left(e^{\sqrt{\xi }}-e^{-\nu }\right)}{\sqrt{\xi }\left(\nu^{2}-\xi \right)\left(e^{2\sqrt{\xi }}-1\right)},$$

and the nondimensionalised open-circuit voltage $\omega_{oc}$ is given by:

$$\omega_{oc}=\ln\left(1+A_{oc}+B_{oc}-\frac{\mu }{\nu^{2}-\xi }\right).$$

Using the separation of variables, we find that $u_{oc}$ is given by:

(13) $$u_{oc}\left(x,t\right)=\sum_{k=0}^{\infty }V_{k}\cos\left(k\pi x\right)e^{-(\xi +\pi^{2}k^{2})t},$$

where the constants $V_{k}$ are of the form:

$$V_{k}=2\int_{0}^{1}\cos\left(k\pi x\right)\left(e^{\omega_{oc}}-f_{oc}\left(x\right)\right).$$

In summary, the analytical solution for the open-circuit model is given by:

(14) $$n\left(x,t\right)=1+A_{oc}e^{\sqrt{\xi }x}+B_{oc}e^{-\sqrt{\xi }x}-\frac{\mu }{\nu^{2}-\xi }e^{-\nu x}+\sum_{k=0}^{\infty }V_{k}\cos\left(k\pi x\right)e^{-(\xi +\pi^{2}k^{2})t}.$$

**Figure 3:**
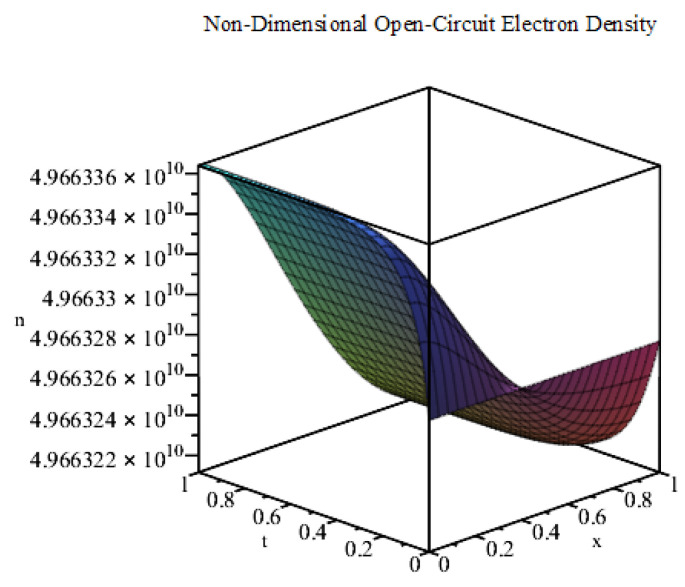
Plot of *n* against *x* and *t* (m=0, open-circuit conditions).

Using the values of the parameters given in Table 1, we plot the exact solution (14) for the first 100 terms of the infinite series in MAPLE (see Figure 3). This solution has a different profile to that of the short-circuit case shown in Figure 2. Here, the electron density is initially at an extremely high magnitude (of the order of $10^{10}$). Then, it shifts slightly to match the exponential source term in accordance with the Neumann boundary conditions. We note that the solution profile of our model is in agreement with that of Gómez and Salvador [17]. We note that the discrepancy between the solutions of the short-circuit and open-circuit is due primarily to the Dirichlet boundary condition for the short-circuit model and the parameter ξ. The short-circuit electron density prescribed at the TCO electrode is small relative to the electron density found for the open-circuit model.

**Figure 4:**
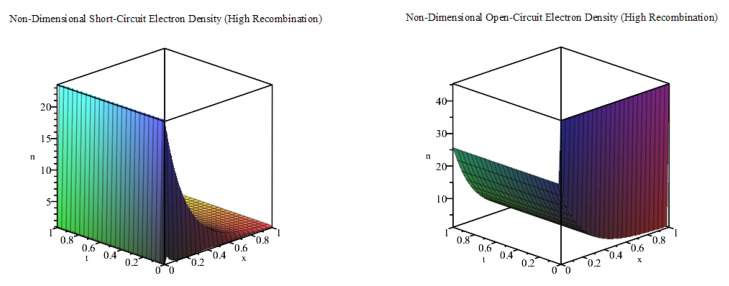
Plot of *n* against *x* and *t* (m=0) for the case of a high recombination effect.

Next, we use $k_{R}=400$ $s^{-1}$, so that $\xi =10^{5}$ (μ and ν have the same values as in Table 1) to plot the short-circuit and open-circuit electron densities, as shown together in Figure 4. As we increase the value of ξ, which results in a higher level of recombination, we find that the discrepancy between the open-circuit and short-circuit electron densities is much smaller. Additionally, the solutions do not vary much from their initial states in comparison to Figure 2 and Figure 3.

In the following section, we consider the nonlinear electron diffusion equation for which m>0.

## 3. Classical Lie Symmetry for the Nonlinear Electron Diffusion Equation

In this section, we seek analytical solutions for particular forms of the nonlinear diffusion equation by using classical Lie symmetry techniques. First, we rewrite Equation (4) in a general form as:

(15) $$\frac{\partial n}{\partial t}=\frac{\partial }{\partial x}\left[D\left(n\right)\frac{\partial n}{\partial x}\right]+G\left(x\right)+R\left(n\right).$$

The method of Lie symmetry was first developed by Sophus Lie in the late 19th Century [20]. This framework offered a systematic approach to finding transformations of variables in a differential equation that lead to a reduction of order (or integrating factors for a first order ODE). Finding these transformations amounts to solving a system of linear differential equations, which is referred to in the literature as classical symmetries. Classical symmetry analysis has been used to analyse diffusion equations for decades. The studies have led to a vast knowledge of particular structures of the diffusion equation that admit classical Lie symmetry (see, for example, [21,22,23]). Nevertheless, many special cases involving spatially dependent source terms have not yet been considered. In this paper, we look for special cases of Equation (15) that may admit analytical solutions by the classical Lie symmetry method.

### 3.1. Classical Lie Symmetry

To find other classical symmetries of diffusion equations that are not covered in [21], we use an algebraic computer package DIMSYM [24]. For Equation (15) in its current form, the only symmetry operator is of the form:

$$\Gamma_{1}=\frac{\partial }{\partial t},$$

which corresponds to an invariant under translation in time. Furthermore, DIMSYM suggests six special cases that may potentially yield further symmetries:

1. D(n)=0,
2. $\frac{dD}{dn}=0$,
3. $\frac{d^{3}D}{dn^{3}}\frac{dD}{dn}D\left(n\right)-2{\left(\frac{d^{2}D}{dn^{2}}\right)}^{2}D\left(n\right)+\frac{d^{2}D}{dn^{2}}{\left(\frac{dD}{dn}\right)}^{2}=0$,
4. $x\frac{dG}{dx}+2G\left(x\right)+2R\left(n\right)=0$,
5. The set $\left\{x\frac{dG}{dx},\frac{dG}{dx},G,1\right\}$ is linearly dependent, or
6. R(n)=0.

Given that this model is based on the assumption of electron diffusing within the conduction band of DSSCs, the diffusion coefficient must not be zero. Assuming $\left\{x\frac{dG}{dx},\frac{dG}{dx},G,1\right\}$ is linearly dependent or R(n)=0 does not admit any new symmetries without also making assumptions about the diffusion coefficient. Note that we consider R(n)=0 as part of other special cases.

#### 3.1.1. Constant Diffusion Coefficient

Here, we suppose that D(n) is a constant. Without loss of generality, we may assume D(n)=1. Equation (15) is therefore of the form:

(16) $$\frac{\partial n}{\partial t}=\frac{\partial^{2}n}{\partial x^{2}}+G\left(x\right)+R\left(n\right).$$

We note that if R(n)=an+b for some constants *a* and *b*, then Equation (16) reduces to a linear partial differential equation, which can be solved by using standard techniques. Generally, Equation (16) does not immediately admit symmetries other than $\Gamma_{1}$ above. Two special cases are:

1. The set $\left\{n\frac{\partial R}{\partial n},\frac{\partial R}{\partial n},R,n,1\right\}$ is linearly dependent or
2. *G* is a constant.

If *G* is a constant, Equation (15) reduces to the case that has already been classified by Dorodnitsyn [22]. We therefore assume that *G* is not a constant and consider recombination terms for *R* that ensure $\left\{n\frac{\partial R}{\partial n},\frac{\partial R}{\partial n},R,n,1\right\}$ is linearly dependent. That is, R(n) is of the form:

- $R\left(n\right)=c_{1}{\left(n+c_{2}\right)}^{c_{3}}+c_{4}n+c_{5}$,
- $R\left(n\right)=c_{1}e^{c_{2}n}+c_{3}n+c_{4}$,
- $R\left(n\right)=c_{1}\ln\left(n+c_{2}\right)+c_{3}n+c_{4}$, or
- $R\left(n\right)=c_{1}\left(n+c_{2}\right)\ln\left(n+c_{2}\right)+c_{3}n+c_{4}$,

where $c_{1},c_{2},c_{3},c_{4}$, and $c_{5}$ are arbitrary constants. Though there are several special cases giving rise to symmetries (found in Table 2), none of these yield analytical solutions unless R(n) is linear.

#### 3.1.2. Particular Diffusion Coefficient

Recall the third order ordinary differential equation for the diffusion coefficient given in Section 3.1,

(17) $$\frac{d^{3}D}{dn^{3}}\frac{dD}{dn}D\left(n\right)-2{\left(\frac{d^{2}D}{dn^{2}}\right)}^{2}D\left(n\right)+\frac{d^{2}D}{dn^{2}}{\left(\frac{dD}{dn}\right)}^{2}=0.$$

We notice that only the power-law and exponential functions for D(n) are able to satisfy Equation (17) (as shown by Edwards [25], for example). Under a power-law diffusivity, the special cases from DIMSYM are that m=−1 and $-\frac{4}{3}$ and $\left\{x\frac{dG}{dx},\frac{dG}{dx},G,1\right\}$ is linearly dependent. Since m≥0 for the study of DSSCs, we assume that $\left\{x\frac{dG}{dx},\frac{dG}{dx},G,1\right\}$ is linearly dependent.

We note that if G(x) is constant, the equation is of the form $n_{t}={\left(D\left(n\right)n_{x}\right)}_{x}+R\left(n\right)$, which has already been extensively studied (in [21,23,26], for example). Clarkson and Mansfield [27] give an account of a wide range of analytical solutions found for this special case. Otherwise, G(x) is of the form:

- $G\left(x\right)=A{\left(x+B\right)}^{C}$,
- $G\left(x\right)=Ae^{Bx}$, or
- G(x)=Aln(x+B),

for some constants *A*, *B*, and *C*. Table 2 contains special cases for (4) and (17) admitting symmetries. Though these special cases for G(x) admit nontrivial symmetries for both power-law and exponential functions of D(n), none of these special cases reduce to analytical solutions unless *G* is a constant.

#### 3.1.3. Particular Source Term

From the fourth special case in Section 3.1, the electron generation G(x) and the recombination R(n) can be shown to have the form:

$$G\left(x\right)=\frac{\lambda }{2}+Ax^{-2},\;R\left(n\right)=-\frac{\lambda }{2},$$

for some constant λ. In this case, Equation (15) reduces to:

$$\frac{\partial n}{\partial t}=\frac{\partial }{\partial x}\left[D\left(n\right)\frac{\partial n}{\partial x}\right]+Ax^{-2},$$

for some constant *A*. This special case admits the symmetry $\Gamma_{2}=x\frac{\partial }{\partial x}+2t\frac{\partial }{\partial t}$, an invariant of which is the Boltzmann similarity variable $s=xt^{-\frac{1}{2}}$. In this case, the above partial differential equation reduces to the ordinary differential equation:

$$-\frac{1}{2}s\frac{dn}{ds}=\frac{d}{ds}\left[D\left(n\right)\frac{dn}{ds}\right]+As^{-2}.$$

However, we find that there are no symmetries that can reduce this equation further under power-law or exponential diffusivities.

### 3.2. Summary

Table 2 summarises all classical Lie symmetries found with DIMSYM [24].

Under these special cases, we obtain invariants of the form F(x,t,n) by solving $X\frac{\partial F}{\partial x}+T\frac{\partial F}{\partial t}+N\frac{\partial F}{\partial n}=0$. For example, we consider the PDE:

(18) $$\frac{\partial n}{\partial t}=\frac{\partial }{\partial x}\left[n^{m}\frac{\partial n}{\partial x}\right]+Ae^{Bx}+\alpha n^{m+1}.$$

From Table 2, we find that the PDE admits the symmetry:

$$\Gamma =\frac{\left(m+1\right)}{Bm}\frac{\partial }{\partial x}-t\frac{\partial }{\partial t}+\frac{n}{m}\frac{\partial }{\partial n}.$$

Upon solving Γ(F)=0, we find the invariants $s=x+\frac{\left(m+1\right)}{Bm}\ln\left(t\right)$ and $\varphi =nt^{\frac{1}{m}}$. Using these invariants, we may reduce (18) to the ODE:

$$\frac{d}{ds}\left[\varphi^{m}\frac{d\varphi }{ds}\right]-\frac{\left(m+1\right)}{Bm}\frac{d\varphi }{ds}+\alpha \varphi^{\left(m+1\right)}+\frac{1}{m}\varphi +Ae^{Bs}=0.$$

This ODE may also be analysed by classical Lie symmetry, and we find that the ODE does not admit more symmetries.

## 4. Nonclassical Lie Symmetry for the Nonlinear Electron Diffusion Equation

Developed in 1969, Bluman and Cole [28] added an additional invariant surface condition, which transformed the linear equations of classical symmetry to nonlinear equations. This approach is now known as nonclassical symmetry or Q-conditional symmetry in the literature. Results on nonclassical symmetry are far more recent, but nevertheless extensive [26,27,29,30,31].

A particular study of nonclassical symmetry developed in 1993 by Nucci [32] is the method of Heir equations, which assumes particular forms for the invariant surface conditions. Recently, Bradshaw-Hajek [33] investigated the partial differential equation:

$$u_{t}={\left[D\left(u\right)u_{x}\right]}_{x}+R\left(x,u\right),$$

for the special cases R(x,u)=Q(u) and R(x,u)=r(x)Q(u). This study was motivated by problems in mathematical biology, and we refer to Joshi and Morrison [34] for a generalised form for R(x,u) with the additional assumption of constant diffusion coefficient (D(u)=D).

### 4.1. Nonclassical Symmetry Analysis for $D\left(n\right)=n^{m}$

To find nonclassical symmetries for a partial differential equation of the form:

$$\frac{\partial n}{\partial t}=\frac{\partial }{\partial x}\left[n^{m}\frac{\partial n}{\partial x}\right]+G\left(x\right)+R\left(n\right),$$

we use MAPLE to compute and expand the second prolongation $\Gamma^{\left(2\right)}$, which is the differential operator given by:

(19) $$\Gamma^{\left(2\right)}=X\frac{\partial }{\partial x}+T\frac{\partial }{\partial t}+N\frac{\partial }{\partial n}+N_{t}^{\left(1\right)}\frac{\partial }{\partial n_{t}}+N_{x}^{\left(1\right)}\frac{\partial }{\partial n_{x}}+N_{xx}^{\left(2\right)}\frac{\partial }{\partial n_{xx}}+N_{xt}^{\left(2\right)}\frac{\partial }{\partial n_{xt}}+N_{tt}^{\left(2\right)}\frac{\partial }{\partial n_{tt}},$$

where subscripts for *n* denote the usual partial derivatives and $N_{x_{i}x_{j}}^{\left(k\right)}$ are the extended infinitesimals. We refer the reader to [25] for details of the differential operator $\Gamma^{\left(2\right)}$.

This step leads to the Lie symbol:

$$\Gamma =X\frac{\partial }{\partial x}+T\frac{\partial }{\partial t}+N\frac{\partial }{\partial n}.$$

Following the nonclassical technique given by Bluman and Cole [28], we also include the invariant surface condition:

(20) $$X\frac{\partial n}{\partial x}+T\frac{\partial n}{\partial t}=N.$$

Adopting a common convention for nonclassical symmetry analysis for evolution equations, we set T=1 so the invariant surface condition (20) can be rewritten as:

$$\frac{\partial n}{\partial t}=N-X\frac{\partial n}{\partial x},$$

leading to the following series of determining equations:

(21) $$n\frac{\partial^{2}X}{\partial n^{2}}-m\frac{\partial X}{\partial n}=0,$$

(22) $$-mn^{m-1}\frac{\partial N}{\partial n}+mn^{m-2}N-2X\frac{\partial X}{\partial n}+2n^{m}\frac{\partial^{2}X}{\partial x\partial n}-u^{m}\frac{\partial^{2}N}{\partial n^{2}}=0,$$

(23) $$\begin{array}{c} -2n^{m+1}\frac{\partial^{2}N}{\partial x\partial n}+n^{m+1}\frac{\partial^{2}X}{\partial x^{2}}-3n\left(G\left(x\right)+R\left(n\right)-\frac{2}{3}N\right)\frac{\partial X}{\partial n}\\ \;-2mn^{m}\frac{\partial N}{\partial x}-2nX\frac{\partial X}{\partial x}+mXN-n\frac{\partial X}{\partial t}=0, \end{array}$$

(24) $$\begin{array}{c} -n^{m+1}\frac{\partial^{x}N}{\partial x^{x}}-2n\left(G(x)+R(n)-N\right)\frac{\partial X}{\partial x}+n\left(G(x)+R(n)\right)\frac{\partial N}{\partial n}\\ \;+n\frac{\partial N}{\partial t}-mN^{2}+\left(m\left(G\left(x\right)+R\left(n\right)\right)-n\frac{\partial R}{\partial n}\right)N-n\frac{dG}{dx}X=0. \end{array}$$

Solving Equation (21) as an ODE for X(n) yields:

$$X\left(x,t,n\right)=f\left(x,t\right)+n^{m+1}g\left(x,t\right),$$

for some arbitrary functions f(x,t) and g(x,t). Upon substituting X(x,t,n), Equation (22) simplifies to:

$$n^{2}\frac{\partial^{2}N}{\partial n^{2}}+mn\frac{\partial N}{\partial n}-mN=2\left(m+1\right)n^{2}\left(\frac{\partial g}{\partial x}n^{m}-f\left(x,t\right)g\left(x,t\right)-g{\left(x,t\right)}^{2}n^{m+1}\right).$$

Given that the parameter *m* is non-negative, the general solution to this equation is given by:

(25) $$\begin{array}{c} N\left(x,t,n\right)=h\left(x,t\right)n+k\left(x,t\right)n^{-m}+\frac{1}{\left(m+1\right)}\frac{\partial g}{\partial x}n^{m+2}\\ \;-\frac{2(m+1)}{\left(m+2\right)}f\left(x,t\right)g\left(x,t\right)n^{2}-\frac{2\left(m+1\right)g{\left(x,t\right)}^{2}}{\left(2m+3)(m+2\right)}n^{m+3}, \end{array}$$

where *h* and *k* are arbitrary functions of *x* and *t*. Splitting (23) and (24) as a polynomial in *n* leads only to the trivial symmetry $\Gamma =\frac{\partial }{\partial t}$ for general source terms G(x) and R(n), as we assume m≥0.

For the special case when m=1, G(x)=x+A, and R(n)=0, the PDE can be written as:

$$\frac{\partial n}{\partial t}=\frac{\partial }{\partial x}\left[n\frac{\partial n}{\partial x}\right]+x+A,$$

which admits the symmetry:

$$\Gamma =\frac{2(x+A)}{3\mu +t}\frac{\partial }{\partial x}+\frac{\partial }{\partial t}+\frac{3n}{3\mu +t}\frac{\partial }{\partial n},$$

for some constant μ. We note that the symmetry is equivalent to the classical symmetries found for this special case (as seen in Table 2). Nevertheless, this symmetry produces the invariant variables $\psi =n{\left(t+3\mu \right)}^{-3}$ and $s=\left(x+A\right){\left(t+3\mu \right)}^{-2}$, leading to the reduction:

$$\frac{d}{d\psi }\left[s\frac{ds}{d\psi }\right]+2s\frac{ds}{d\psi }-3\psi +s=0.$$

There are no classical symmetries that may reduce this equation further.

### 4.2. Nonclassical Symmetry Analysis for $D\left(n\right)=e^{mn}$

To find nonclassical symmetries for the partial differential equation,

(26) $$\frac{\partial n}{\partial t}=\frac{\partial }{\partial x}\left[e^{mn}\frac{\partial n}{\partial x}\right]+G\left(x\right)+R\left(n\right),$$

we use MAPLE to compute and expand the second prolongation $\Gamma^{\left(2\right)}$ with the same setup as with the power law diffusion coefficient. The determining equations are:

(27) $$-e^{mn}\left(m\frac{\partial X}{\partial n}-\frac{\partial^{2}X}{\partial n^{2}}\right)=0,$$

(28) $$-me^{mn}\frac{\partial N}{\partial n}-2X\frac{\partial X}{\partial n}+2e^{mn}\frac{\partial^{2}X}{\partial n\partial x}-e^{mn}\frac{\partial^{2}N}{\partial n^{2}}=0,$$

(29) $$-2e^{mn}\frac{\partial^{2}N}{\partial x\partial n}+e^{mn}\frac{\partial^{2}X}{\partial x^{2}}+\left[2N-3(G(x)+R(n))\right]\frac{\partial X}{\partial n}-2me^{mn}\frac{\partial N}{\partial x}+mXN-2X\frac{\partial X}{\partial x}-\frac{\partial X}{\partial t}=0,$$

(30) $$\begin{array}{c} -e^{mn}\frac{\partial^{2}N}{\partial x^{2}}+2\left(N-G\left(x\right)-R\left(n\right)\right)\frac{\partial X}{\partial x}+\left(G\left(x\right)+R\left(n\right)\right)\frac{\partial N}{\partial n}+\frac{\partial N}{\partial t}-mN^{2}\\ \;+\left[m\left(G\left(x\right)+R\left(n\right)\right)-\frac{dR}{dn}\right]N-\frac{dG}{dx}X=0. \end{array}$$

The general solution for Equation (27) is given by:

(31) $$X\left(x,t,n\right)=f\left(x,t\right)e^{mn}+g\left(x,t\right),$$

for arbitrary functions f(x,t) and g(x,t). Equation (28) implies:

(32) $$N\left(x,t,n\right)=\frac{\frac{\partial f}{\partial x}-f{\left(x,t\right)}^{2}}{m}e^{mn}+h\left(x,t\right)e^{-mn}-2f\left(x,t\right)g\left(x,t\right)n+k\left(x,t\right),$$

for some arbitrary functions h,k of *x* and *t*. Splitting each term for *n* in (29) and (30) leads to the trivial symmetry $\Gamma =\frac{\partial }{\partial t}$ for general source terms G(x) and R(n). We find that the special cases yielding a nontrivial symmetry are precisely those in which *G* is constant, which has already been studied (in [35], for example).

## 5. Numerical Results and Discussion

In this paper, we present a nonlinear diffusion model for electron density in the conduction band of the nano-porous semiconductor ${\mathrm{TiO}}_{2}$ in a DSSC. This equation admits an exact analytical solution under linear diffusion (m=0). For the general case of m>0, both classical and nonclassical Lie symmetry analyses indicated no new analytical solutions for the physically relevant special cases of the partial differential equations studied here. As a result, we proceeded to obtain solutions numerically.

Using a standard forward time central space finite difference scheme in MATLAB (detailed in Maldon et al. [36]), we provided numerical solutions to Equation (4) for both power-law and exponential diffusion coefficients under short-circuit conditions. We simulated for dimensional time t∈[0,1].

**Figure 5:**
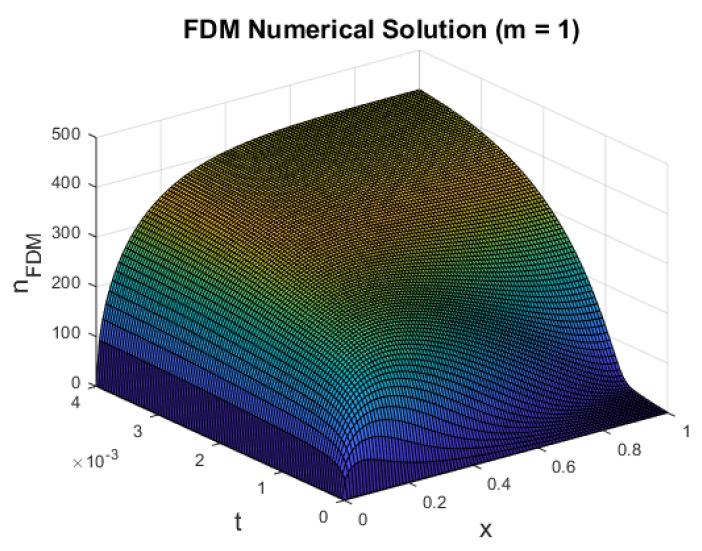
Plot of *n* against *x* for dimensional t=1 and m=1.

In Figure 5, we plot the numerical solution to the following partial differential equation,

$$\frac{\partial n}{\partial t}=\frac{\partial }{\partial x}\left[n\frac{\partial n}{\partial x}\right]+\mu e^{-\nu x}-\xi n\left(n-1\right),$$

where we assume m=1 and subject to short-circuit boundary conditions. We note that D(n)=n was found to best resemble the nonlinear diffusion of electron density present in the semiconductor conduction band [6,8,37]. The solution profile in Figure 5 is similar to that of the linear case (m=0) shown in Figure 2. However, the magnitude of the electron density differs by a factor of $O(10^{2})$. Since *m* in the diffusion coefficient $D\left(n\right)=n^{m}$ determines the strength of electron trap in the ${\mathrm{TiO}}_{2}$ structure, we expected a lower value of the electron density for the case of m=1 as this corresponds to a higher effect of the trap, preventing electron transport throughout the DSSC.

**Figure 6:**
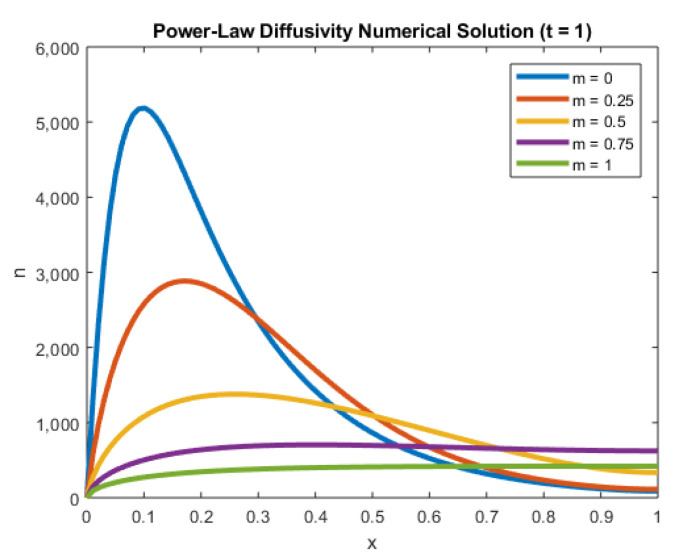
Plot of *n* against *x* at t=1 for several values of *m*.

In Figure 6, we elucidate the influence of *m* on the electron density as given in Equation (1). In particular, we consider $m=0,\frac{1}{4},\frac{1}{2},\frac{3}{4}$, and m=1.

From the figure, we see that for lower power-law diffusivities, the electron density reached the steady-state slower. This case could cause potential stability issues in numerical calculations, owing to the higher overall electron densities. Conversely, the higher power-law diffusivities (stronger traps) in the ${\mathrm{TiO}}_{2}$ nano-porous structure led to significantly lower electron density. For higher values of m, the solutions also reached the steady-state faster.

**Figure 7:**
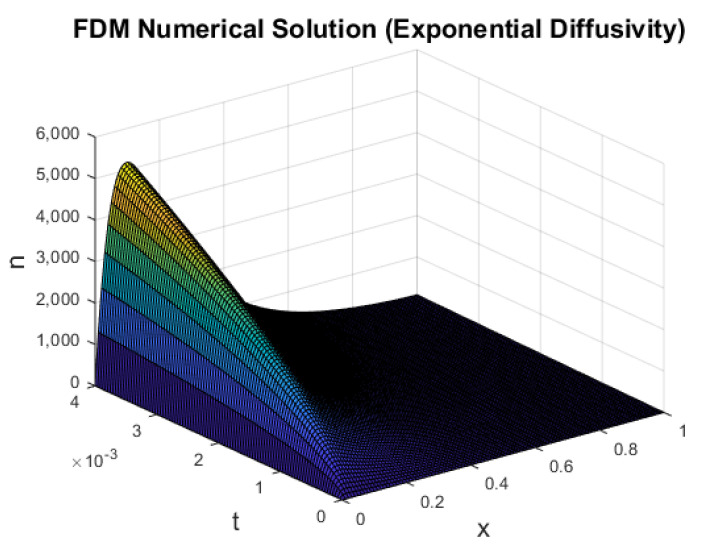
Plot of *n* against *x* and *t* (exponential diffusivity).

To investigate the effect of an exponential diffusivity, in Figure 7, we plot the numerical solution n(x,t) for the following equation,

(33) $$\frac{\partial n}{\partial t}=\frac{\partial }{\partial x}\left[e^{mn}\frac{\partial n}{\partial x}\right]+\mu e^{-\nu x}-\xi e^{mn}\left(n-1\right),$$

for which the diffusion coefficient is $D\left(n\right)=e^{mn}$. We note that the recombination term also took the same exponential coefficient (as shown in [8]). To better resemble the power-law diffusivity and avoid stability issues, *m* was assumed to take the form $m=\frac{\ln(n_{eq})}{n_{eq}}$. Under the data in Table 1, this meant that $m=O(10^{-21})$. The numerical solution shown in Figure 7 fundamentally resembles the power-law diffusivities presented in Figure 6.

**Figure 8:**
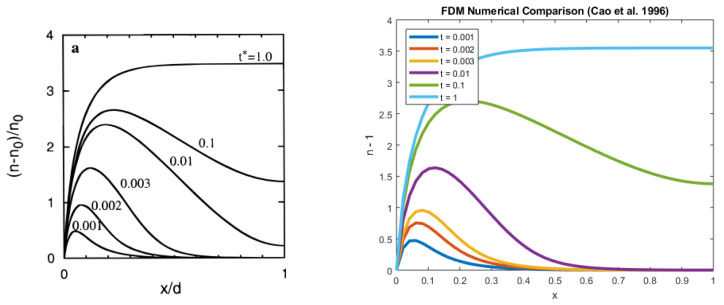
Comparison of the results with Cao et al. [6] (Reprinted with permission from (Cao, F., Oskam, G., Meyer, G.J. and Searson, P.C., 1996. Electron transport in porous nanocrystalline ${\mathrm{TiO}}_{2}$ photoelectrochemical cells. The Journal of Physical Chemistry, 100(42), pp.17021-17027.). Copyright 1996 American Chemical Society).

In Figure 8, we use parameter values consistent with Cao et al. [6] to solve Equation (4) and find good agreement with their results. In comparison to our studies, their diffusion coefficient was significantly higher, and the recombination term was omitted. These densities nevertheless compared favourably well as the recombination parameter ξ was very small ($\xi =10^{-5}$ in our case).

### Efficiency Calculations

Using the formulae in Section 1.2 and Section 1.3, we computed the efficiency using different diffusivity functions D(n) as shown in Table 3. For each D(n), we simulated the numerical solution to time $t=10^{3}$ and computed the short-circuit current density $J_{sc}$, the open-circuit voltage $V_{oc}$, and efficiency η.

From Table 3, we see that linear diffusion outperformed all other diffusion coefficients. For the diffusivity, $D\left(n\right)=n,\sqrt{n},n^{2}$, we see that they hindered the electron density so strongly that the resulting short-circuit current density $J_{sc}$ and the efficiency were adversely affected. From the values of $J_{sc}$, $V_{oc}$, and η presented in Table 3, we found that the linear diffusion model with a constant D(n) was in agreement with data of DSSCs available in the literature [1].

In summary, this paper explored the use of nonlinear diffusion equations to model electron density within the nano-porous semiconductor of DSSCs. We showed that classical and nonclassical Lie symmetry analysis indicated no physically relevant analytical solutions for the general form of the nonlinear diffusion Equation (1). However, using a finite difference method to solve (1) for particular cases of diffusion coefficients and recombination terms, we saw good agreement between our solutions and those given in the literature.

The role of nonlinear diffusion is shown by Figure 6, in which the electron density is greatly affected by the power-law. Given the depth of active traps within the ${\mathrm{TiO}}_{2}$ network [8], as well as a nonlinear recombination term [10], we see the importance of using nonlinear PDEs to model diffusion in DSSCs.

Finally, we comment that in order to develop more realistic mathematical models for DSSCs, the role of the electrolytes and the efficiency of the counter electrode should be included in the model to better describe the photoelectrochemical activity in DSSCs. 

## Figures and Tables

**Table 1:** Parameter values for the DSSCs used in diffusion models.

Parameter	Value	Reference
$D_{0}$	$10^{-11}\;m^{2}\cdot s^{-1}$	[8]
$\alpha_{ab}$	$10^{5}\;m^{-1}$	[12]
*d*	$5\times 10^{-5}\;m$	[8]
$k_{R}$	$4\times 10^{-8}\;s^{-1}$	[8]
$n_{eq}$	$10^{22}\;m^{-3}$	[16]
φ	$10^{21}\;m^{-2}\cdot s^{-1}$	[17]
μ	$2.5\times 10^{6}$	
ν	5	
ξ	$10^{-5}$	

**Table 2:** Classical Lie symmetries found for special cases of Equation (4).

D(n)	G(x)	R(n)	*X*	*T*	*N*
1	Ax+B	$n^{\frac{1}{3}}$	*x*	2t	3n
1	$Ax^{-2}$	$\alpha e^{\beta n}$	$\frac{x}{2}$	*t*	$-\frac{n}{\beta }$
1	−2αln(x+B)+C	αln(n+β)	x+B	2t	2(n+β)
1	$Ae^{Bx^{2}+Cx}$	αnln(n)+γn	$\frac{1}{Ce^{4Bt}}$	0	$-\frac{\left(C+2Bt\right)}{Ce^{4Bt}}$
1	$Ax^{-4}$	$\alpha n^{2}$	*x*	2t	−2n
$n^{m}$	$A{\left(x+B\right)}^{C}$	$\alpha n^{\frac{C}{\left(C+2\right)}}$	(m+1)(x+B)	(2−Cm)t	(C+2)n
$n^{m}$	$Ae^{Bx}$	$\alpha n^{m+1}$	$\frac{\left(m+1\right)}{Bm}$	−t	$\frac{n}{m}$
$n^{m}$	Aln(x+B)+C	$-\frac{A(m+1)}{2}\ln\left(n\right)$	(m+1)(x+B)	2t	2n
$e^{mn}$	$Ax^{B}$	$\alpha e^{\frac{Bmn}{B+2}}$	*x*	−Bt	$\frac{B+2}{m}$
$e^{mn}$	$Ae^{Bx}$	$\alpha e^{mn}$	1	−Bt	$\frac{B}{m}$
$e^{mn}$	Aln(x+B)+C	$-\frac{Amn}{2}$	x+B	0	$\frac{2}{m}$
D(n)	$Ax^{-2}$	0	*x*	2t	0

**Table 3:** Calculated performance characteristics for different diffusion coefficients.

D(n)	η	$J_{\mathrm{sc}}\left({\mathrm{Am}}^{-2}\right)$	$V_{\mathrm{oc}}\left(V\right)$
1	7.0569	133.8545	0.6322
$\sqrt{n}$	0.5054	10.8597	0.5673
*n*	0.1247	2.8818	0.5330
$n^{2}$	0.0298	0.7452	0.4980
$e^{\frac{\ln(n_{eq})}{n_{eq}}n}$	7.0569	133.8545	0.6322

## Abbreviations

The following abbreviations are used in this manuscript:

DSSC	Dye-sensitized solar cell
IPCE	Incident photon to current efficiency
PDE	Partial differential equation
